# Unsupervised machine learning identifies two distinct posterior tibial slope clusters largely independent of Coronal Plane Alignment of the Knee in osteoarthritic knees

**DOI:** 10.1002/jeo2.70945

**Published:** 2026-10-13

**Authors:** Dinko Vidović, Dejan Blažević, Josip Baković, Fabijan Čukelj, Tin Karakaš, Borna Vojvodić, Anja Barešić, Tomislav Šmuc, David Glavaš Weinberger

**Affiliations:** ^1^ Department of Traumatology Sestre Milosrdnice University Hospital Center Zagreb Croatia; ^2^ School of Medicine University of Zagreb Zagreb Croatia; ^3^ School of Dental Medicine University of Zagreb Zagreb Croatia; ^4^ School of Medicine Catholic University of Croatia Zagreb Croatia; ^5^ Faculty of Health Studies University of Zagreb Zagreb Croatia; ^6^ Department for Machine Learning Ruđer Bošković Institute Zagreb Croatia

**Keywords:** clustering, Coronal Plane Alignment of the Knee, knee surgery, machine learning, posterior tibial slope

## Abstract

**Purpose:**

Coronal‐plane classifications such as the Coronal Plane Alignment of the Knee (CPAK) classification support individualized total knee arthroplasty (TKA) planning but do not account for sagittal‐plane morphology, particularly the posterior tibial slope (PTS). This study aimed to determine whether unsupervised machine learning (k‐means clustering) could identify naturally occurring PTS phenotypes in osteoarthritic knees and to evaluate their association with CPAK classification and joint line obliquity (JLO).

**Methods:**

This retrospective radiographic cohort study included 3337 osteoarthritic knees from 1746 patients. PTS was measured on lateral knee radiographs in all 3337 knees, using the posterior tibial cortex and the medial tibial plateau as reference lines. Full‐length standing radiographs were available for 464 knees; in these knees, the medial proximal tibial angle (MPTA) and the lateral distal femoral angle (LDFA) were measured to calculate the arithmetic hip–knee–ankle angle (aHKA) and JLO, and each knee was classified according to CPAK. K‐means clustering was used to identify PTS subgroups without predefined thresholds, and cluster validity was assessed using the elbow method and silhouette analysis. Associations between PTS clusters, CPAK phenotypes and radiographic parameters were analysed using the *χ* ^2^ test, adjusted standardized residuals, Fisher's exact test and correlation analysis.

**Results:**

The median PTS was 5.6° (interquartile range 3.4°–7.7°). K‐means clustering identified two PTS clusters: a shallow‐slope cluster centred at 3.56°, comprising 55.91% of the knees and a steep‐slope cluster centred at 8.52°, comprising 44.09%. No individual CPAK phenotype was significantly overrepresented within either cluster. When phenotypes were grouped according to joint line orientation, however, apex‐distal phenotypes (I–III) were more frequently assigned to the steep‐slope cluster, whereas apex‐neutral phenotypes (IV–VI) were more frequently assigned to the shallow‐slope cluster. JLO was significantly higher in the shallow‐slope cluster and PTS showed a modest inverse correlation with JLO.

**Conclusions:**

Unsupervised k‐means clustering identified two distinct sagittal PTS phenotypes in osteoarthritic knees. PTS varied largely independently of the individual CPAK phenotypes, although an association with JLO was observed. Sagittal morphology may therefore provide complementary information beyond coronal alignment classifications for the individualized planning of knee surgery.

**Level of Evidence:**

Level IV.

## Graphical abstract

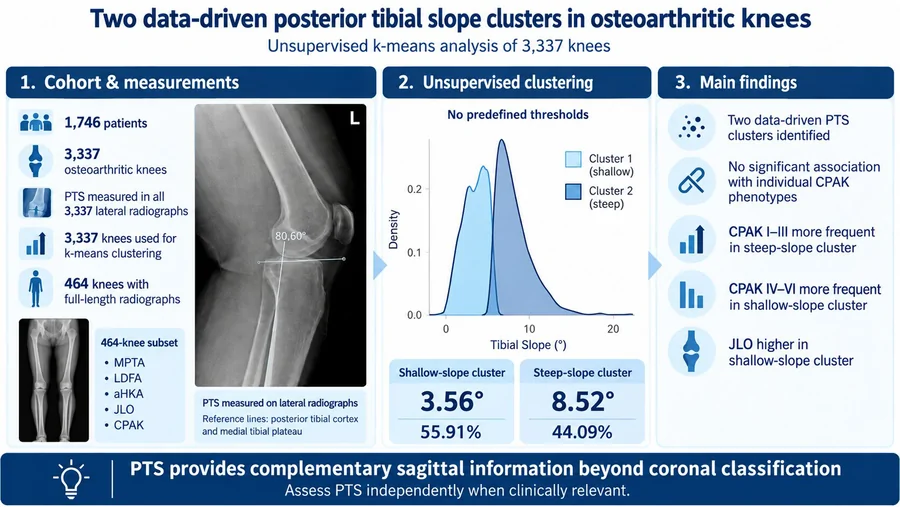

**Abbreviations**
aHKA: arithmetic hip–knee–ankle angle
AP: anteroposterior
CPAK: Coronal Plane Alignment of the Knee
HKA: hip–knee–ankle angle
ICC: intraclass correlation coefficient
IQR: interquartile range
JLO: joint line obliquity
LDFA: lateral distal femoral angle
MPTA: medial proximal tibial angle
PACS: Picture Archiving and Communication System
PTS: posterior tibial slope
TKA: total knee arthroplasty

## INTRODUCTION

Accurate characterization of knee morphology is fundamental to the planning of knee reconstructive procedures, including revision anterior cruciate ligament (ACL) reconstruction, around‐the‐knee osteotomy (AKO) and total knee arthroplasty (TKA), particularly when patient‐specific correction or alignment targets are pursued. Such planning requires the assessment of multiple anatomical and biomechanical parameters, including overall limb alignment, femoral and tibial coronal orientation, rotational alignment, joint‐line orientation, anterior and posterior femoral offset, patellofemoral alignment and the posterior tibial slope (PTS).

Phenotype‐based classification systems have been developed to characterize constitutional knee anatomy more precisely. Hirschmann et al. proposed the functional knee phenotype classification, which recognizes the wide variability in native lower‐limb alignment beyond the traditional mechanical alignment concept [15]. Among these systems, the Coronal Plane Alignment of the Knee (CPAK) classification is increasingly used in TKA planning. It combines the hip–knee–ankle angle (HKA), calculated from the medial proximal tibial angle (MPTA) and the lateral distal femoral angle (LDFA), with joint line obliquity (JLO) to define nine coronal phenotypes [28].

However, CPAK does not account for sagittal or axial morphology. Three‐dimensional computed tomography (CT) studies have demonstrated that coronal classifications capture only a small proportion of the substantial variability in sagittal and axial anatomy, including PTS, rotational parameters and trochlear morphology [6, 13, 21]. Contemporary phenotype concepts and consensus frameworks have therefore moved toward multiplanar assessment of patient‐specific morphology [11, 14, 36]. CPAK should thus be regarded as a pragmatic coronal reference rather than a comprehensive knee phenotype, and PTS requires independent assessment.

An increased PTS has been associated with ACL graft failure, and recent studies have identified an elevated risk at slopes exceeding approximately 10°–12° [5, 10]. PTS is therefore increasingly considered during the preoperative assessment for revision ACL surgery and slope‐modifying AKO, although no consensus exists on the value that should be regarded as pathological, particularly in the setting of AKO [26, 37]. In TKA, PTS is determined by the proximal tibial resection and influences knee kinematics, ligament balance and flexion‐gap behaviour [12, 32, 40]. Restoring the native PTS to within 4° has been associated with better clinical outcomes after robot‐assisted TKA with functional alignment [3], whereas a steeper‐than‐native cut may cause instability and a shallower cut may reduce the range of motion and produce gap imbalance [33, 35]. Nevertheless, no universal PTS target has been established, and conventional alignment techniques do not accurately reproduce the high interindividual variability of the native PTS [2].

Previous attempts to integrate PTS into coronal phenotyping have relied on predefined thresholds [32]. Like the CPAK categories themselves, such fixed cut‐offs simplify interpretation but are inherently rigid and may overlook the natural distribution of PTS and nonlinear relationships with coronal morphology. Unsupervised machine learning can identify intrinsic subgroups without imposing predefined thresholds and has increasingly been used to characterize knee kinematics and alignment patterns [1, 31].

However, whether PTS forms naturally occurring, data‐driven subgroups and how such subgroups relate to coronal phenotypes, has not been established. This question may be clinically relevant, as coronal phenotypes differ in their reconstructive behaviour, with some being more difficult to reproduce anatomically than others, and unrecognized sagittal variation may contribute to this [7, 18, 19]. Clarifying whether PTS differs systematically between CPAK phenotypes may therefore improve the understanding of the interaction between coronal and sagittal morphology and inform individualized surgical planning.

This study aimed to characterize the distribution of PTS in osteoarthritic knees, to determine whether unsupervised k‐means clustering could identify data‐driven PTS subgroups and to evaluate their association with CPAK classification and JLO. The prespecified hypothesis was that distinct PTS clusters could be identified and that their distribution would differ according to coronal knee morphology.

## METHODS

### Study design and patient selection

This study was conducted as a retrospective cohort study. Prior to its initiation, approval was granted by the Ethics Committee of the Sestre Milosrdnice University Hospital Center (ethical approval number: 251‐29‐11/3‐24‐04), authorizing access to the medical records and radiographic data of eligible patients. Patient assessment included the collection of demographic data, specifically age and sex. Standard anteroposterior (AP), lateral lower‐leg and full‐length standing radiographs were reviewed for the measurement of MPTA, LDFA and PTS. PTS was measured on lateral knee radiographs that showed at least 15 cm of the proximal tibia, according to the technique described by Hohmann et al., which uses the posterior tibial cortex as the vertical reference axis and the medial tibial plateau as the horizontal reference axis [17] (Figure 1). This technique has been reported to show the highest interrater reliability among the commonly applied methods for assessing PTS [9].

**Figure 1:**
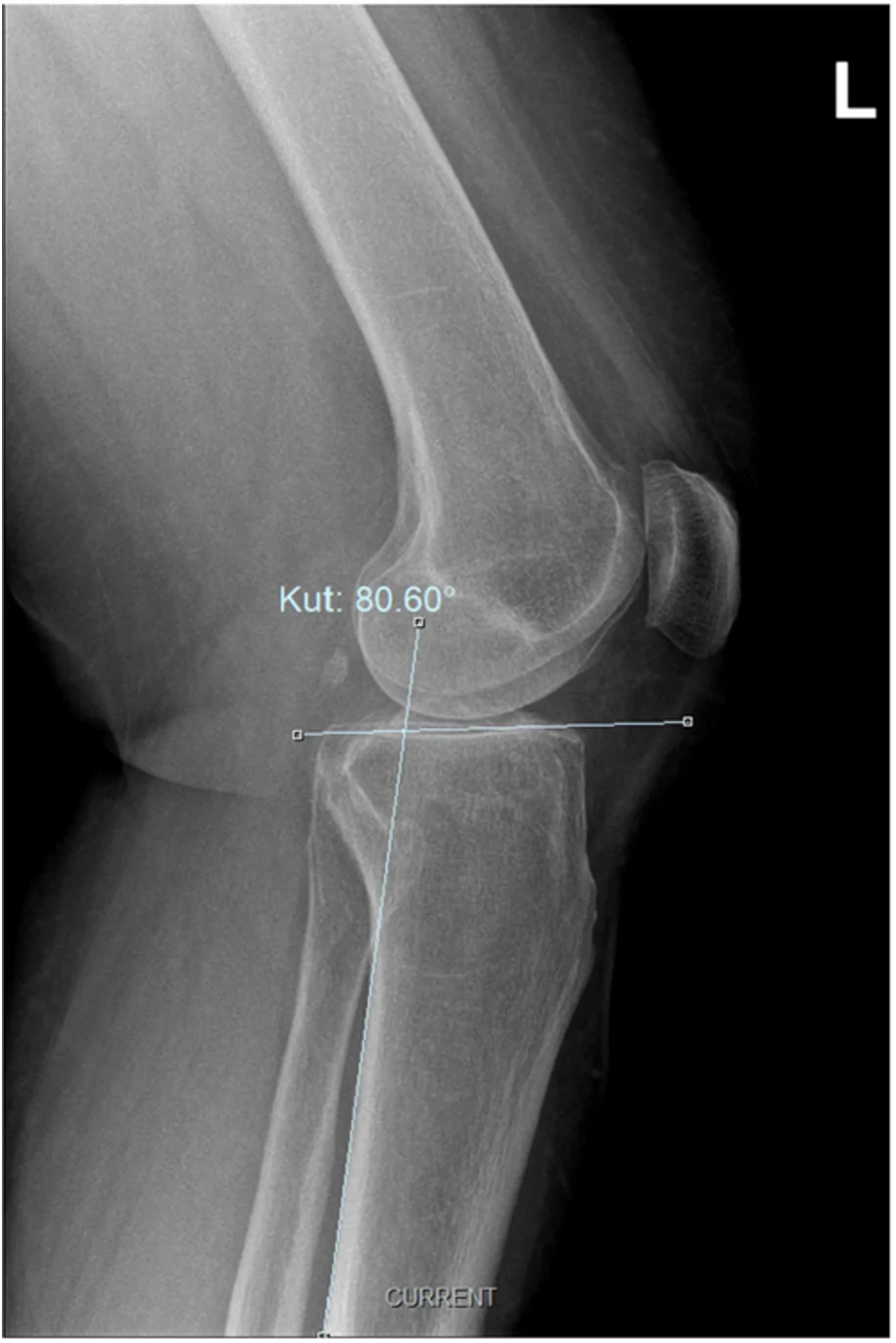
Measurement of PTS on lateral knee radiographs using the method described by Hohmann et al. The posterior tibial cortex and the medial tibial plateau serve as the reference lines for the measurement. ‘Kut’ means angle in Croatian; PTS, posterior tibial slope.

Patients with osteoarthritic knees were included if lateral knee radiographs suitable for PTS measurement were available. The radiographs were required to show at least 15 cm of the proximal tibia and to allow identification of the posterior tibial cortex. The exclusion criteria were post‐traumatic osteoarthritis, a history of corrective osteotomy and the presence of tumours in the periarticular region of the knee. Patients were also excluded if the required radiographs were not available in the hospital's Picture Archiving and Communication System (PACS).

### Measurement protocol

Full‐length standing radiographs were available for 464 knees. Five independent examiners measured MPTA and LDFA on these radiographs, as well as PTS on the corresponding lateral lower‐leg radiographs. A calibration phase was performed on the first 100 full‐length standing radiographs, during which all five examiners independently measured MPTA, LDFA and PTS in order to assess inter‐rater reliability using intraclass correlation coefficients (ICCs).

After high agreement between the observers had been confirmed, the remaining radiographs were distributed equally among the five examiners for the final analysis. Each examiner performed all assigned measurements independently on three separate occasions, and the arithmetic mean of the repeated measurements was used for the statistical analysis.

The arithmetic HKA (aHKA) was calculated as the difference between MPTA and LDFA, whereas JLO was calculated as their sum. Based on these values, each knee was subsequently classified according to the CPAK classification system.

### Statistical analysis

Statistical and graphical analyses were performed using RStudio 2024.12.0 + 467 (Posit PBC). A *p* value < 0.05 was considered statistically significant.

ICCs were used to assess both the inter‐rater and the intra‐rater reliability of the MPTA, LDFA and PTS measurements. Inter‐rater reliability was evaluated during the calibration phase, in which all five observers independently measured the same initial set of 100 radiographs. After high inter‐rater agreement had been confirmed, the remaining radiographs were distributed equally among the observers, and intra‐rater reliability was then assessed from three repeated measurements performed independently by each observer on separate occasions.

The normality of the distributions was tested using the Lilliefors variant of the Kolmogorov–Smirnov test. Numerical variables are reported as mean ± standard deviation or as median with interquartile range (IQR), as appropriate. Normally distributed variables were compared using parametric tests, whereas variables that were not normally distributed were compared using non‐parametric tests, namely, the Mann–Whitney *U* test and Spearman's *ρ*. Effect sizes were calculated using Cliff's *δ* for variables that were not normally distributed and Cohen's *d* for normally distributed variables. Categorical variables are presented as frequencies and percentages.

Unsupervised k‐means clustering was used to identify naturally occurring subgroups of knees based on PTS, without applying predefined threshold values. K‐means clustering partitions observations into groups with similar characteristics while maximizing the differences between groups. The optimal number of clusters was determined using the elbow method and silhouette analysis: The elbow method identifies the point at which additional clusters no longer substantially improve model fit, whereas silhouette analysis evaluates cluster cohesion and separation, with higher silhouette widths indicating better‐defined clusters. Clustering was performed using Euclidean distance metrics.

The sample size was determined by the number of eligible radiographs available for analysis. A conventional power calculation was not applicable to the unsupervised k‐means clustering procedure, as this method does not test a prespecified null hypothesis. For the primary comparison between the nine CPAK phenotypes and the two PTS clusters, a sensitivity analysis was performed using G*Power 3.1.9.6 (Heinrich‐Heine‐Universität). With 464 knees, eight degrees of freedom, *α* = 0.05 and 80% statistical power, the available sample was sufficient to detect an association corresponding to Cohen's *w* ≥ 0.18.

The *χ* ^2^ test was used to compare the distribution of PTS clusters across the CPAK phenotypes. When a significant overall association was identified, adjusted standardized residuals were calculated for each cell of the contingency table to determine which observed frequencies differed significantly from those expected under the assumption of independence. Positive residuals indicated overrepresentation and negative residuals underrepresentation of individual CPAK phenotypes within specific PTS clusters or sex categories. Absolute residual values (*Z* values) greater than 1.96 were considered statistically significant (*p* < 0.05).

## RESULTS

### Study population

In total, 3337 knees from 1746 patients met the inclusion criteria and were available for PTS analysis. The PTS measurements of the entire cohort were used for the unsupervised k‐means clustering analysis. Corresponding full‐length standing radiographs of sufficient quality for the measurement of MPTA and LDFA were available for 464 knees (13.9% of the total cohort); these measurements were used to calculate aHKA and JLO and to assign each knee to a CPAK phenotype. Accordingly, this subset of 464 knees constituted the study population for the analyses of the relationship between PTS clusters and CPAK classification (Figure 2).

**Figure 2:**
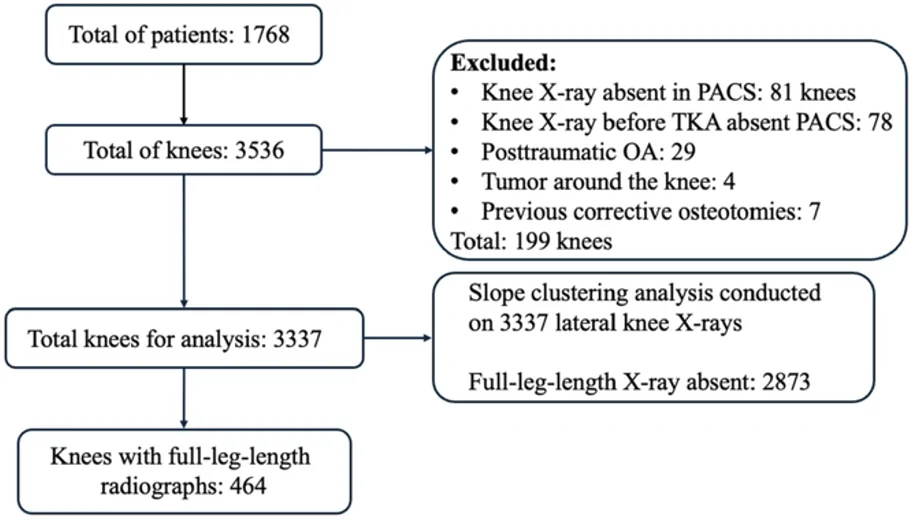
Flowchart of the inclusion criteria and of the knees available for each analysis. OA, osteoarthritis; PACS, Picture Archiving and Communication System; TKA, total knee arthroplasty.

The median age of the patients was 69 years (IQR 63–73). MPTA and PTS were not normally distributed and are therefore reported as median (IQR): MPTA was 86.6° (84.0°–92.2°), and PTS was 5.6° (3.4°–7.7°). LDFA was 88.64° ± 3.47°, aHKA was −2.32° ± 6.78°, and JLO was 175.21° ± 4.68°. Inter‐rater reliability was assessed on the first 100 radiographs, which were measured by all five examiners; MPTA, LDFA and PTS showed high inter‐rater reliability (ICC ≥ 0.8; *p* < 0.001), indicating strong and consistent agreement between the examiners.

After the calibration phase, the remaining radiographs were allocated equally among the five examiners for the definitive analysis, and each examiner performed three independent measurement sessions of all assigned radiographs. Intra‐rater reliability was high for all assessed parameters, with ICC values exceeding 0.95 (*p* < 0.001) for the MPTA, LDFA and PTS measurements.

### CPAK distribution

The most frequent phenotype was CPAK I (34.9%, *n* = 168), followed by CPAK IV (15.3%, *n* = 71) and CPAK III (15.0%, *n* = 65). CPAK II was present in 14.3% of the knees (*n* = 62), CPAK VI in 9.5% (*n* = 44) and CPAK V in 8.3% (*n* = 39). The least frequent phenotypes were CPAK IX (1.2%, *n* = 6) and CPAK VII and VIII (0.6% each, *n* = 3).

### PTS clustering

PTS values did not follow a normal distribution (Lilliefors *p* < 0.001) and appeared as a skewed, double‐peaked curve on the kernel density plot (Figure 3), suggesting the presence of two subpopulations within the cohort.

**Figure 3:**
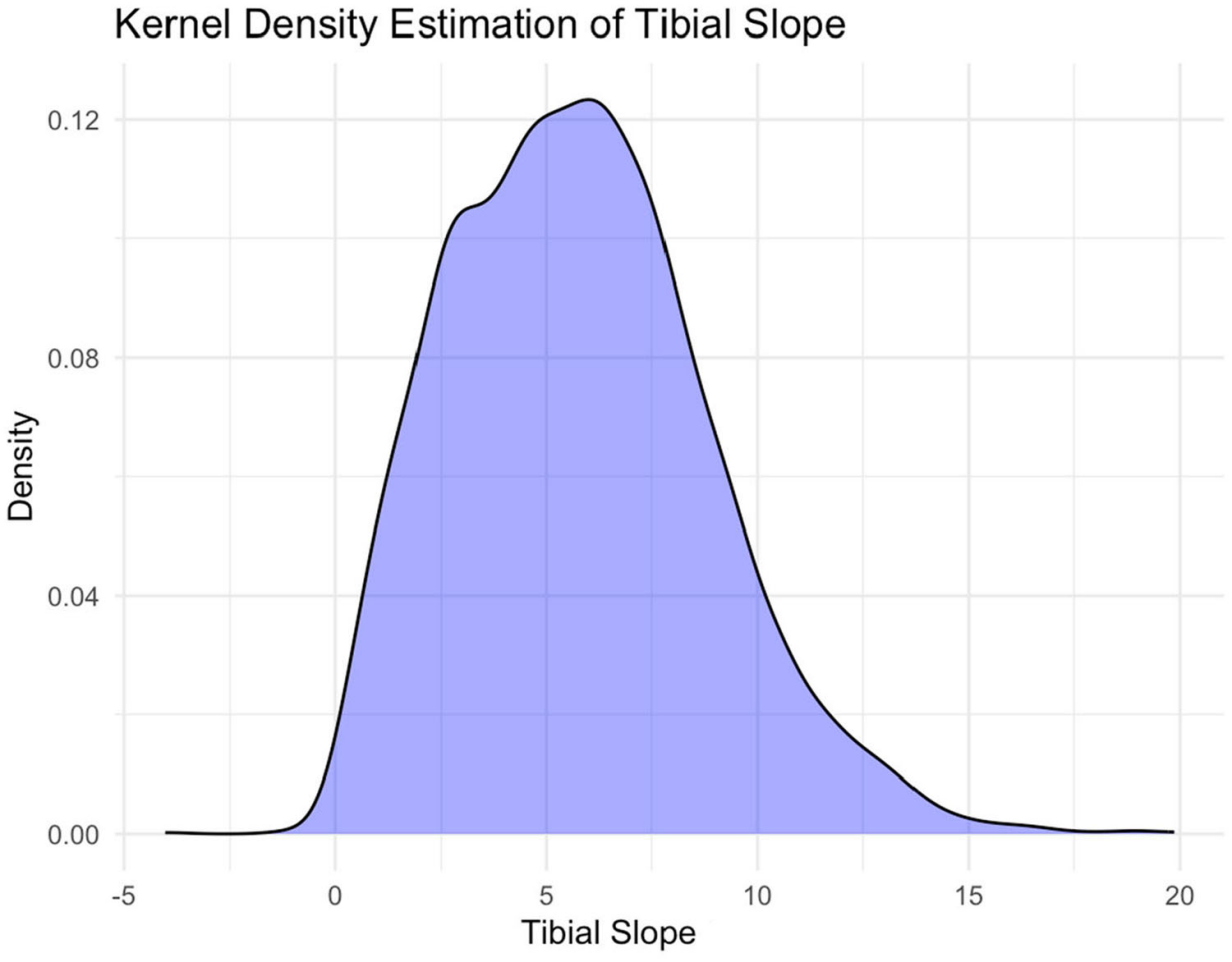
Kernel density estimation of the posterior tibial slope, demonstrating a non‐normal, asymmetric distribution with a secondary density feature.

The PTS values of all 3337 knees were analysed using k‐means clustering, and both the elbow method and silhouette analysis supported a two‐cluster solution.

The overall mean silhouette width for *k* = 2 was 0.56, indicating moderate cluster separation. The shallow‐slope cluster comprised 55.91% of the knees (*n* = 1866), with a centroid of 3.56° and a mean silhouette width of 0.60, whereas the steep‐slope cluster comprised 44.09% of the knees (*n* = 1471), with a centroid of 8.52° and a mean silhouette width of 0.52. PTS values remained non‐normally distributed within both clusters (Lilliefors *p* < 0.001). The median PTS was 3.74° (IQR 2.42°–4.92°) in the shallow‐slope cluster and 8.02° (IQR 7.00°–9.48°) in the steep‐slope cluster, and the distributions differed significantly between the clusters (Mann–Whitney *U* test, *p* < 0.001; Cliff's *δ* = −1) (Figure 4).

**Figure 4:**
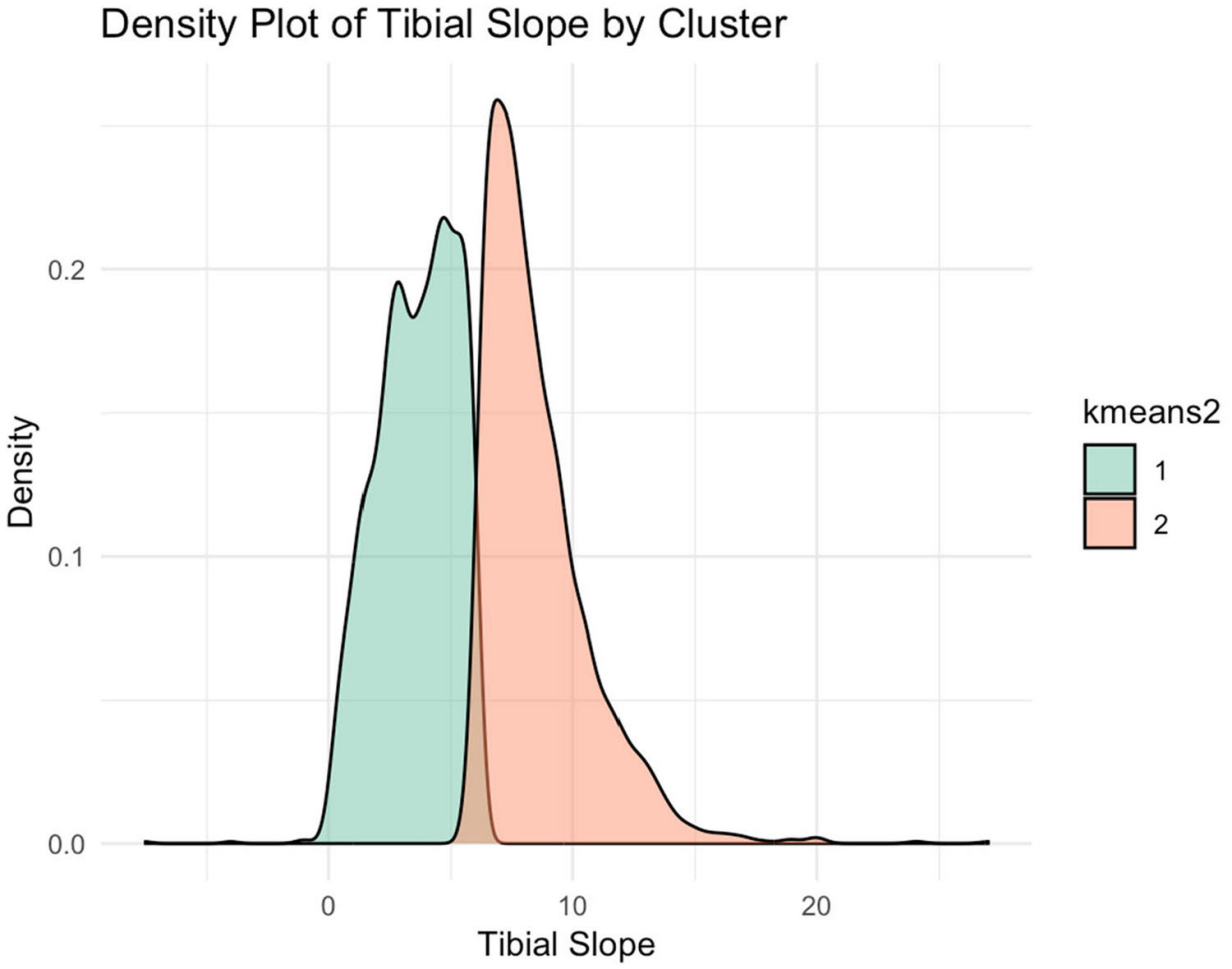
Kernel density plots of the posterior tibial slope according to k‐means cluster assignment. Cluster 1 represents the shallow‐slope cluster and Cluster 2 the steep‐slope cluster. The separation between the distributions reflects the partition generated by the k‐means algorithm.

### Comparison of PTS clusters and CPAK

The distribution of PTS clusters was compared across the nine CPAK phenotypes. A *χ* ^2^ test with Monte Carlo simulation demonstrated a statistically significant overall association between CPAK phenotype and PTS cluster membership (*χ* ^2 ^= 16.41, *p* < 0.05). However, the cell‐wise analysis of adjusted standardized residuals did not identify a statistically significant overrepresentation of either the shallow‐slope or the steep‐slope cluster within any individual CPAK phenotype (Table 1).

**Table 1:** Comparison of PTS clusters and CPAK phenotypes.

CPAK group	Cluster	Number of knees	Frequency (%)	*Z* score	Significance (*p*)
I	Shallow	73	30.7	−1.51^a^	>0.05
	Steep	95	42.0	1.54^a^	>0.05
II	Shallow	35	14.7	0.51	>0.05
	Steep	27	11.9	−0.52	>0.05
III	Shallow	27	11.3	−1.14^a^	>0.05
	Steep	38	16.8	1.17^a^	>0.05
IV	Shallow	44	18.5	1.28^a^	>0.05
	Steep	28	12.4	−1.30^a^	>0.05
V	Shallow	23	9.7	0.70	>0.05
	Steep	16	7.1	0.72	>0.05
VI	Shallow	28	11.8	1.39^a^	>0.05
	Steep	16	7.1	−1.42^a^	>0.05
VII	Shallow	2	0.8	0.38	>0.05
	Steep	1	0.4	0.39	>0.05
VIII	Shallow	2	0.8	0.38	>0.05
	Steep	1	0.4	0.39	>0.05
IX	Shallow	4	1.7	−0.04	>0.05
	Steep	4	1.7	0.04	>0.05

Abbreviations: CPAK, Coronal Plane Alignment of the Knee; PTS, posterior tibial slope.

a |*Z*| > 1 was used to select CPAK phenotypes for further analysis.

Descriptively, CPAK I and III included a higher proportion of knees belonging to the steep‐slope cluster (centroid 8.52°), whereas CPAK IV and VI included a higher proportion of knees belonging to the shallow‐slope cluster (centroid 3.56°). To further investigate overarching trends related to JLO, the CPAK phenotypes were grouped according to joint line orientation, with CPAK I–III classified as apex distal and CPAK IV–VI as apex neutral. Fisher's exact test demonstrated a statistically significant difference in the distribution of PTS clusters between these groups (*p* < 0.01): knees with an apex‐distal joint line were significantly more frequently assigned to the steep‐slope cluster, whereas knees with an apex‐neutral joint line were significantly more frequently assigned to the shallow‐slope cluster (*Z* = ±3.13, *p* < 0.01) (Table 2). PTS values did not differ significantly between male and female patients (*p* = 0.269).

**Table 2:** Comparison of PTS values and clusters across apex distal JLO CPAK Groups (I–III) and apex neutral CPAK groups (IV–VI).

CPAK grouped	Number of knees	Frequency (%)	Median PTS (IQR)	Cluster	Number of knees per cluster	Within group frequency (%)	*Z* score*	*p* value
CPAK I–III	295	63.6	6.44 (4.74–8.83)	Shallow	135	45.8	−3.13	<0.01
				Steep	160	54.2	3.13	
CPAK IV–VI	155	33.4	5.19 (3.83–6.88)	Shallow	95	61.3	3.13	<0.01
				Steep	60	38.7	−3.13	

Abbreviations: CPAK, Coronal Plane Alignment of the Knee; IQR, interquartile range; JLO, joint line obliquity; PTS, posterior tibial slope.

*|*Z*|> 2.58 indicates a significance level of *p* < 0.01.

### Comparison of numerical variables

JLO was significantly higher in the shallow‐slope cluster (176° ± 4.39°) than in the steep‐slope cluster (174° ± 4.78°; *p* < 0.001; Cohen's *d* = 0.43; 95% confidence interval [CI] 0.25–0.62). MPTA was also significantly higher in the shallow‐slope cluster (87.3°, 84.5°–89.4°) than in the steep‐slope cluster (86.3°, 82.6°–89.0°; *p* < 0.05), although the effect was moderate (Cliff's *δ* = 0.16; 95% CI 0.07–0.27).

Correlation analysis revealed a significant inverse correlation between PTS and JLO (*ρ* = −0.300; *p* < 0.001). All other significant correlations resulted from the variables being derived from one another.

## DISCUSSION

The key finding of this study is that unsupervised k‐means analysis partitioned the continuous distribution of PTS into two data‐driven clusters, centred at 3.56° and 8.52° and representing shallow and steep slopes, respectively. Furthermore, PTS cluster membership was largely independent of the individual CPAK phenotypes. An exploratory secondary analysis based on joint line orientation demonstrated a significant difference between the apex‐distal and apex‐neutral CPAK groups, and PTS and JLO showed a modest inverse correlation. These findings indicate that sagittal tibial morphology contains information that is not adequately represented by the individual coronal CPAK categories.

This interpretation is consistent with recent evidence demonstrating that PTS is only weakly captured by coronal phenotype classifications. Hiyama et al. reported that PTS was largely independent of the CPAK classification and concluded that sagittal and coronal alignment should be assessed separately [16]. Similarly, Franceschetti et al. found no significant differences in PTS between CPAK categories, despite variability across functional tibial phenotypes [8]. Three‐dimensional CT data have shown that aHKA and JLO explain only a limited proportion of the variability in tibial slope [27], and recent EOS‐based analyses have demonstrated inherent three‐dimensional limitations of the CPAK framework [38]. The limited relationship between PTS and the individual CPAK phenotypes observed in the present study is therefore in line with these previous reports. The present study adds a methodological perspective by demonstrating that, when PTS is analysed without predefined thresholds, two reproducible population‐level clusters emerge that remain largely uncaptured by the individual CPAK categories.

Substantial interindividual variability in PTS has previously been demonstrated, with reported values covering a wide anatomical range [22, 30, 39]. Reducing sagittal tibial morphology to a single population mean or to an arbitrary threshold may therefore inadequately represent this variability. Morrisey et al. proposed adding PTS to the CPAK framework using an 8° threshold [32]. The present analysis approached the same problem from a different perspective: rather than imposing a predetermined cut‐off, unsupervised clustering allowed the observed distribution itself to determine the partition. The resulting cluster centroids of approximately 3.6° and 8.5° are notable, as they lie close to values that have previously been considered clinically relevant. The modest relationship observed between PTS and JLO further suggests that a population‐level coronal–sagittal association exists, although its clinical implications require further investigation.

Sagittal tibial morphology influences native knee biomechanics and has been associated with ACL injury and with knee function after ACL reconstruction [17, 29]. The assessment of an excessive PTS has therefore become increasingly relevant in revision ligament surgery, and a recent expert consensus supports considering slope correction in selected patients undergoing revision ACL reconstruction [37]. In this context, the identification of shallow‐ and steep‐slope clusters provides an additional framework for describing population‐level variation in sagittal tibial anatomy and may facilitate future investigation of procedure‐specific biomechanical and clinical consequences.

PTS also remains relevant in TKA, because modification of the tibial slope can influence ligament tension, flexion‐gap behaviour, tibiofemoral kinematics, contact loading and prosthetic mechanics [4, 12, 23, 25, 34, 35, 40]. However, no universally accepted sagittal target is applicable to all TKA designs or alignment philosophies, and the optimal management of PTS depends on patient anatomy, implant characteristics and the selected surgical strategy [24, 35, 40]. The present findings nevertheless have a practical implication for preoperative assessment: An identical sagittal target may represent substantially different alterations of the native morphology in knees located near the shallow‐slope and the steep‐slope centroid. A tibial slope of 3°–5°, for example, would approximate the native morphology of many knees within the shallow‐slope cluster but would represent a substantially greater reduction from the native PTS in knees approaching the steep‐slope centroid. The present study does not establish whether either strategy provides superior clinical outcomes, but it illustrates why the patient‐specific PTS should be known before deciding whether, and to what extent, sagittal anatomy should be modified. Moreover, because the individual CPAK phenotypes did not reliably predict PTS cluster membership, coronal phenotype classification cannot substitute for direct sagittal assessment. This may be particularly relevant when PTS is expected to influence implant mechanics and ligament balance in TKA, or when ligament reconstruction or slope‐modifying osteotomy is planned.

From a methodological perspective, the use of single‐variable k‐means clustering was intentional, as the objective was to characterize the intrinsic variation in PTS without introducing predefined thresholds or additional anatomical variables. Although clustering methods are more frequently applied to multidimensional datasets [1, 20, 31], the present analysis produced a two‐cluster solution with a silhouette width of 0.56, indicating moderate cluster separation. This supports the usefulness of the partition for describing population‐level PTS heterogeneity but does not establish the existence of discrete clinical phenotypes. Future machine‐learning approaches in larger cohorts, integrating sagittal, coronal, axial and patellofemoral morphology, may provide a more comprehensive multidimensional representation of the knee phenotype.

Several limitations should be acknowledged. First, the retrospective single‐centre design limits the external validity of the findings. Although PTS clustering was performed in the complete cohort of 3337 knees, the full‐length standing radiographs required for CPAK classification were available for only 464 knees (13.9%). The analyses examining the relationship between PTS clusters and CPAK were therefore performed in this smaller subset and may be susceptible to selection bias. Nevertheless, the sensitivity analysis demonstrated that 464 knees provided 80% power to detect an overall CPAK–PTS cluster association corresponding to Cohen's *w* ≥ 0.18, which still allows most subtle associations to be detected. Second, clustering was intentionally based on a single continuous variable. K‐means partitions the observed distribution and does not establish that the identified groups represent discrete biological populations or clinically applicable treatment thresholds; external validation of the identified clusters in independent cohorts is therefore warranted. Third, CPAK was used as the coronal reference framework, whereas other sagittal, axial and patellofemoral morphological parameters were not incorporated into the analysis. Finally, clinical outcomes were not assessed; the present study therefore cannot determine whether membership in a particular PTS cluster is associated with the surgical strategy, postoperative stability, range of motion or patient‐reported outcomes. Prospective studies integrating multidimensional knee morphology with procedure‐specific clinical outcomes are required to determine whether these data‐driven PTS patterns have predictive or therapeutic value.

The results of the present study may have several clinical implications. In this setting, unsupervised machine learning methods offer an objective framework for identifying anatomical patterns without relying on preconceived thresholds. Because all measurements in this study were obtained from routinely available long‐leg and lateral knee radiographs, this approach may facilitate the identification of patient subgroups and support individualized surgical planning with standard imaging modalities.

## CONCLUSION

Unsupervised k‐means analysis identified two data‐driven PTS clusters, centred at 3.56° and 8.52°, within the PTS distribution of osteoarthritic knees. PTS cluster membership was largely independent of the individual CPAK phenotypes, although the modest inverse relationship between PTS and JLO suggests a subtle population‐level interaction between sagittal and coronal morphology. These findings support the view that PTS provides complementary sagittal information that is not captured by coronal phenotype classification alone and that it should therefore be assessed independently when clinically relevant.

## AUTHOR CONTRIBUTIONS

Dinko Vidović, Anja Barešić and David Glavaš Weinberger were responsible for the study conception and design. David Glavaš Weinberger, Borna Vojvodić, Josip Baković, Tin Karakaš and Fabijan Čukelj Jr. performed the radiographic measurements of the medial proximal tibial angle, LDFA and PTS. Data collection, formal analysis and investigation were performed by David Glavaš Weinberger, Josip Baković, Fabijan Čukelj Jr., Tin Karakaš, Borna Vojvodić, Anja Barešić and Tomislav Šmuc. The first draft of the manuscript was written by Dinko Vidović and David Glavaš Weinberger. Dinko Vidović, David Glavaš Weinberger, and Dejan Blažević were responsible for manuscript review and editing. Dinko Vidović supervised the study. All authors read and approved the final version of the manuscript.

## FUNDING INFORMATION

The authors have no funding to report.

## CONFLICT OF INTEREST STATEMENT

The authors declare no conflicts of interest.

## ETHICS STATEMENT

The study was performed in accordance with the ethical standards as laid down in the 1964 Declaration of Helsinki and its later amendments. Approval was granted by the Ethics Committee of the Sestre Milosrdnice University Hospital Center (ethical approval number: 251‐29‐11/3‐24‐04). The requirement for informed consent was waived by the institutional ethics committee due to the retrospective nature of the study and the use of anonymized radiographic data.

## Data Availability

The data that support the findings of this study are available from the corresponding author, David Glavaš Weinberger, upon reasonable request.
